# NF-κB-induced NOX1 activation promotes gastric tumorigenesis through the expansion of SOX2-positive epithelial cells

**DOI:** 10.1038/s41388-019-0702-0

**Published:** 2019-01-30

**Authors:** Kanae Echizen, Keigo Horiuchi, Yayoi Aoki, Yoichi Yamada, Toshinari Minamoto, Hiroko Oshima, Masanobu Oshima

**Affiliations:** 1 Division of Genetics, Cancer Research Institute, Kanazawa University, Kanazawa, 920-1192 Japan; 2 AMED-CREST, AMED, Japan Agency for Medical Research and Development, Tokyo, 100-0004 Japan; 3 Faculty of Electrical and Computer Engineering, Institute of Science and Engineering, Kanazawa University, 920-1192, Kanazawa, Japan; 4 Division of Translational and Clinical Oncology, Cancer Research Institute, Kanazawa University, Kanazawa, 920-8640 Japan; 5 WPI-Nano Life Science Institute, Kanazawa University, Kanazawa, 920-1192 Japan

**Keywords:** Cancer microenvironment, Gastric cancer

## Abstract

We previously showed that NADPH oxidase organizer 1 (Noxo1), a component of NADPH oxidase 1 (NOX1), is a TNF-α-induced tumor-promoting factor in gastric tumorigenesis. However, the mechanism of NOX1-induced reactive oxygen species (ROS) signaling for the gastric tumorigenesis has not been understood. Here, we showed that expression of NOX1 complex components, including *Noxo1*, but not other NOX family members was significantly upregulated in both mouse models for gastritis and gastric tumors, which was associated with increased ROS levels. We also found that NF-κB directly regulated *NOXO1* expression in TNF-α-stimulated gastric cancer cells, suggesting that inflammation induces NOX1 complex activation through TNF-α/NF-κB pathway. Notably, in situ hybridization indicated that *Noxo1* mRNA was detected in proliferating cells of gastritis and gastric tumors, and pharmacological inhibition of NOX activity significantly suppressed the proliferation of MKN45 gastric cancer cells and gastric hyperplasia of *K19-C2mE* mice. These results suggest that NOX1/ROS signaling has an important role in increased proliferation of stomach epithelial cells in the inflamed mucosa. Moreover, we found that expression of SOX2, a marker of gastric epithelial stem cells, was increased by NOX1/ROS signaling. Furthermore, disruption of *Noxo1* in *K19-C2mE* mice significantly suppressed gastritis-associated metaplastic hyperplasia, a potent preneoplastic lesion, which was associated with decreased number of SOX2-positive cells. These results indicate that inflammation-induced *Noxo1* expression is responsible for development of metaplastic hyperplasia in the stomach through an increase in SOX2-expressing undifferentiated epithelial cells. Therefore, inhibition of the NOX1/ROS signaling pathway is a possible strategy for prevention and therapy for gastric cancer development.

## Introduction

Stomach cancer is the fourth most frequent cancer in the world, and the second leading cause of mortality by malignancy [1]. Infection with *Helicobacter pylori* is tightly associated with gastric cancer development. Chronic inflammation caused by *H. pylori* infection has been thought to contribute to epithelial transformation [2]. Inflammatory responses promote cancer development by many mechanisms [3]; for example, induction of cyclooxygenase (COX)-2, an enzyme that synthases prostaglandin, is expressed in the inflamed mucosa, which has a crucial role in tumorigenesis by activating downstream prostaglandin E_2_ (PGE_2_) signaling [4]. In gastric cancer tissues, COX-2 is indeed widely upregulated, and the COX-2 and downstream PGE_2_ pathway is continuously activated [5, 6].

Previously, we constructed mouse model (*K19-C2mE* mice) that develops gastritis-associated metaplastic hyperplasia caused by transgenic expression of both *Ptgs2* and *Ptges* [7]. The stomach phenotype of *K19-C2mE* mice appears to be similar to that of spasmolytic polypeptide-expressing metaplasia (SPEM), which is characteristic to *Helicobacter*-infected gastric mucosa and is considered as a potent precursor of gastric cancer [8–10]. Polymorphism in the gene encoding proinflammatory cytokine TNF-α increased the risk of stomach cancer [11, 12], and we also showed that TNF-α is required for the development of metaplastic hyperplasia in *K19-C2mE* mouse stomach [8]. Furthermore, TNF-α expression in macrophages promotes tumorigenesis in the of *Gan* mouse stomach, a model of inflammation-associated stomach cancer caused by transgenic activation of PGE_2_ pathway and Wnt signaling [13]. Using the *Gan* mouse model, we identified NADPH oxidase organizer 1 (Noxo1) as a TNF-α-dependent tumor-promoting factor for gastric tumorigenesis [6, 13].

NOX1 produces reactive oxygen species (ROS). NOXO1 is one of components forming NOX1 complex. Although ROS-induced oxidative stress is generally considered as detrimental to cells, it has been shown that ROS signaling promotes tumorigenesis by induction of DNA mutations as well as by activating oncogenic pathways [14]. In the stomach, oxidative stress is increased by *H. pylori* infection-associated chronic inflammation because of ROS generation, which is a possible important tumor-promoting factor for gastric tumorigenesis [10]. TNF-α-induced NOXO1 is required for NOX1 assembly and activation [15], and it has been shown that NOX1/ROS pathway is a key role in cellular transformation by suppression of protein phosphatases resulting in constitutive activation of oncogenic tyrosine kinase pathways [16, 17]. NOX1 expression is shown to be required for RAS-induced cellular transformation [18, 19]. Furthermore, NOX1 is activated in intestinal tumor cells by Wnt-induced Rac activation, another component of NOX1 complex, which further induces expression of stem cell signature in cancer cells [20]. These results indicate that NOX1-induced ROS production is important for cancer development; however, the mechanisms of NOX1/ROS signaling for inflammation-associated gastric tumorigenesis have not been fully clarified.

Here, we examined the role of NOX1 in stomach tumorigenesis using mouse models as well as gastric cancer cells. We found that *Noxo1* expression is found in proliferating epithelial cells through activation of NF-κB pathway. Furthermore, inhibition of NOX complex suppressed the proliferation of gastric epithelial cells. Moreover, the stem cell-associated molecule SOX2 was upregulated in a NOX-dependent mechanism, potentially causing increased proliferation of gastric epithelial cells. Finally, the disruption of *Noxo1* in *K19-C2mE* mice significantly suppressed gastritis-associated metaplastic hyperplasia. These results indicate the role of Noxo1-dependent NOX1/ROS signaling in gastric tumorigenesis, making this step an effective target for preventing gastric tumorigenesis.

## Results

### The expression of Nox1 complex in gastritis and gastric tumors

**Fig. 1:**
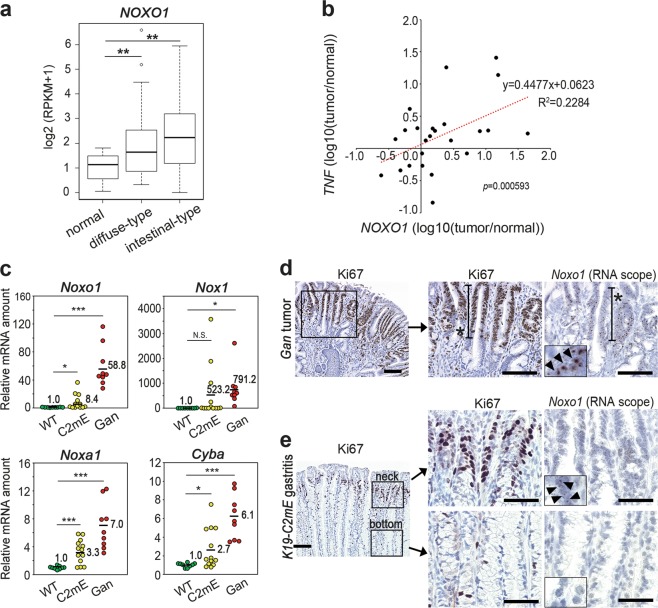
The expression of NOX1 complex components in an inflammation-dependent mechanism. **a** The *NOXO1* mRNA expression in human gastric cancers. The box signifies the upper and lower quartiles. The medians are represented by lines within the box. **p *<* 0.01. **b** The correlation of the relative expression of TNF-α (*TNF*) and *NOXO1* in human gastric cancer to that of adjacent normal stomach is shown. Each dot indicates the result of an individual patient set. **c** The relative mRNA levels of the NOX1 complex genes *Noxo1*, *Nox1*, *Noxa1*, and *Cyba* in wild-type (WT) mouse stomach, *K19-C2mE* mouse gastritis (C2mE) and *Gan* mouse gastric tumors (Gan) are shown with mean values. *p < 0.05; ***p < 0.001; N.S. not significant**. d**, **e** Representative photographs of Ki67 staining (left and center) and in situ hybridization (RNAscope) for *Noxo1* mRNA (right) in the *Gan* mouse gastric tumors (**d**) and *K19-C2mE* mouse gastritis mucosa (**e**). Asterisks in **d** indicate proliferating zone. The insets indicate enlarged images, and arrowheads indicate positive signals. Scale bars, 100 µm (**d** and **e**, left) and 50 µm (**e**, center and right)

We previously reported that *Noxo1* expression is induced in gastric tumors by TNF-α signaling and is important for the maintenance of tumorigenicity of human stomach cancer cells [13]. We confirmed that *Noxo1* expression is significantly upregulated in intestinal-type as well as diffuse-type human stomach cancer using a public database (Fig. 1a) [21]. Furthermore, expression of *NOXO1* in human gastric cancer was positively correlated with those of TNF-α significantly (Fig. 1b). We next examined the NOX1 complex components in the mouse model stomachs. Notably, expression of *Nox1*, *Noxa1*, *Cyba* encoding p22phox as well as *Noxo1* was increased significantly in both mouse models for gastric tumors and gastritis in comparison to wild-type mouse stomach, which suggest the inflammation-dependent activation of the NOX1 pathway (Fig. 1c). Among other NOX family members, expression of *Nox2*, *Duox1* and *Duox2* was increased significantly in mouse gastric tumors; however, only *Duox2* showed gastritis-associated induction (Supplementary Fig. 1). These results are consistent with the report, which show that the NOX family members, NOX1 and DUOX2, are activated in the stomach by inflammatory stress [22]. However, the induction level in the tumors was significantly higher in *Nox1* than in *Duox2* (791.2-fold vs. 2.2-fold, respectively), so we focused on NOX1 complex in this study.

We next examined the cell types expressing *Noxo1* by an RNAscope in situ hybridization analysis. In *Gan* mouse tumors, *Noxo1* mRNA was detected in the tumor cells in the proliferating area (Fig. 1d). Notably, in the *K19-C2mE* mouse stomach, *Noxo1* mRNA was detected at the gland neck of hyperplastic lesions where Ki67-positive proliferating cells were clustered, but *Noxo1* mRNA signal was not detected at the gland bottom, where Ki67 staining was negative (Fig. 1e). These results indicate that *Noxo1* is induced in the proliferating epithelial cells in gastric tumors as well as in non-tumorous gastritis-associated hyperplasia, suggesting that NOX1 complex is important for epithelial proliferation of neoplastic and preneoplastic cells.

### Induction of NOXO1 expression by the TNF-α and NF-κB pathway

**Fig. 2:**
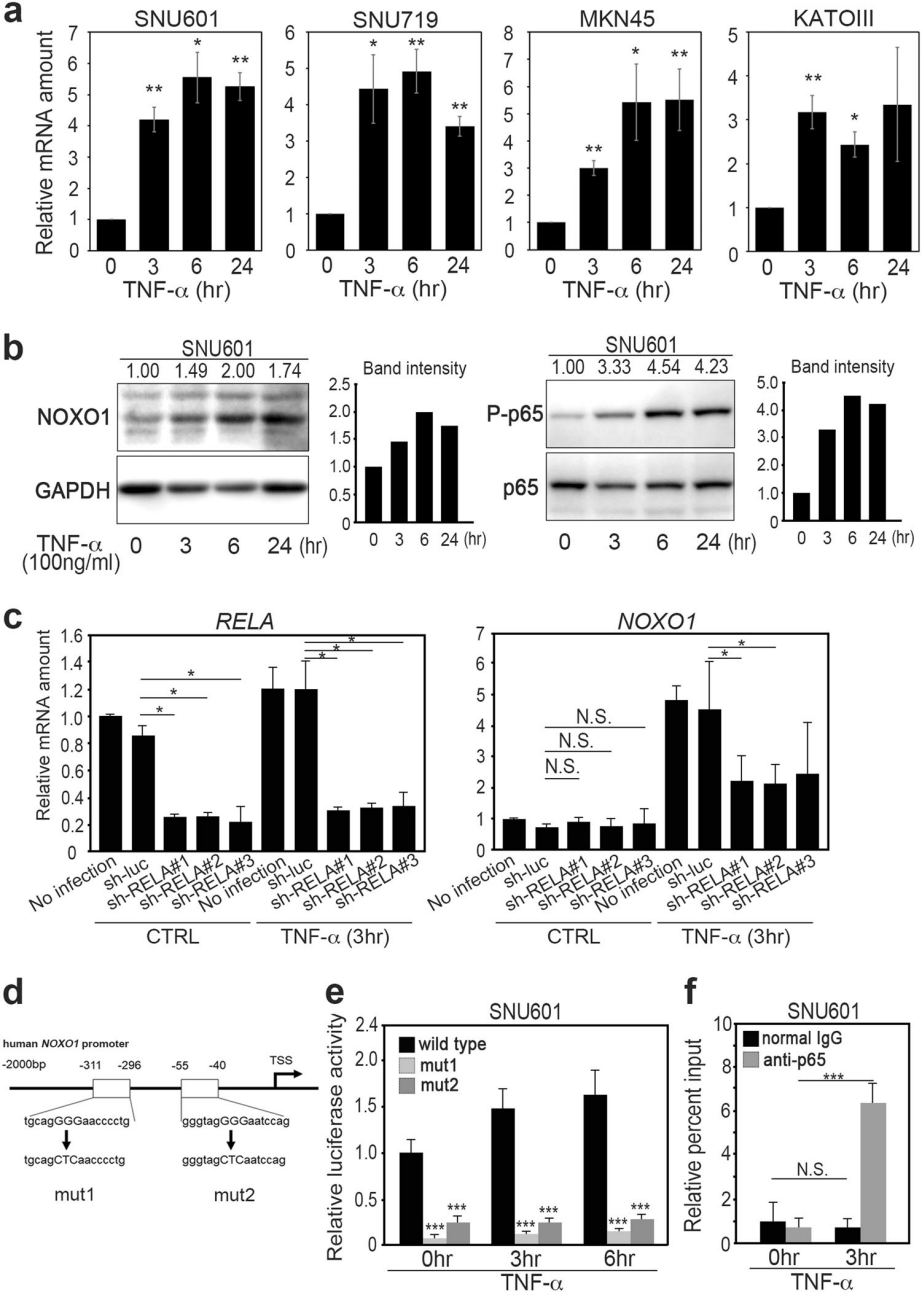
The induction of *NOXO1* expression in gastric cancer cells by TNF-α/NF-κB pathway. **a** The relative mRNA levels of *NOXO1* in the TNF-α-stimulated gastric cancer cells SNU601, SNU719, MKN45, and KATOIII are shown (mean ± s.d.). *p < 0.05; **p < 0.01. **b** Immunoblotting analyses for NOXO1 (left) and phosphorylated p65 (right) in the SNU601 cells after TNF-α stimulation at the indicated time. The relative band intensities to 0 h of TNF-α stimulation are indicated at the top of the panels, along with graphs after normalization with *GAPDH* (left) or p65 (right). **c** The relative mRNA levels of *RELA* (left) and *NOXO1* (right) normalized with the levels of *GAPDH* in the TNF-α-stimulated or unstimulated SNU719 cells are shown (mean ± s.d.). *p < 0.05; N.S. not significant. **d** A schematic view of the *NOXO1* promoter regions (from −2000 bp to the transcription start site [TSS]) showing two putative NF-κB binding sites, indicated by boxes. Mut1 and mut2 vectors contain mutations at either of the two NF-κB binding sites. These fragments were used for a luciferase reporter assay. **e** The relative luciferase activities in SNU601 cells after TNF-α stimulation at 0, 3, and 6 h are shown (mean ± s.d.). ***p < 0.001. **f** The relative percent inputs of ChIP-based real-time PCR results for *NOXO1* promoter in SNU601 cells after TNF-α stimulation at 0 and 3 h are shown (mean ± s.d.). ***p < 0.001; N.S. not significant

We next examined the mechanism of the *NOXO1* induction by TNF-α using human gastric cancer cells. The *NOXO1* mRNA levels were significantly increased in SNU601, SNU719, MKN45 and KATOIII cells by TNF-α stimulation for 3 h, and the levels were maintained for 24 h (Fig. 2a). TNF-α triggers activation of NF-κB. We therefore examined public databases and found that the NF-κB pathway is activated in human gastric cancer, gastritis, and intestinal metaplasia (Supplementary Table 1). Consistently, immunoblotting results indicated that the TNF-α-induced NOXO1 expression was associated with an increased level of phosphorylated p65 (P-p65) of NF-κB in both SNU601 and SNU719 cells, although the P-p65 level was decreased to the basal level at 6 h in SNU719 cells (Fig. 2b, Supplementary Fig. 2a). These results suggest an important role of NF-κB in *NOXO1* induction in TNF-α-stimulated cells. To assess this possibility, we introduced shRNA for *RELA* encoding p65 to SNU719 cells, which resulted in the significant suppression of *RELA* expression (Fig. 2c, left). Notably, the inhibition of *RELA* expression by shRNA significantly suppressed the TNF-α-induced *NOXO1* upregulation (Fig. 2c, right).

We next examined the role of NF-κB in *NOXO1* mRNA transcription by construction of luciferase reporter vectors using wild-type and mutant *NOXO1* gene promoter that contained two putative NF-κB binding sites (Fig. 2d). In two mutant reporter vectors, a mutation was introduced at either of the two NF-κB binding sites. The transfection of the wild-type reporter vector to SNU601 and SNU719 cells significantly increased the luciferase activity by TNF-α stimulation (Fig. 2e, Supplementary Fig. 2b). In contrast, the activity of luciferase was significantly lower in the cells that were transfected with mutant reporter than in the wild-type vector-transfected cells in both cell lines. Furthermore, we confirmed by chromatin immunoprecipitation (ChIP)-based PCR assay that NF-κB binds to the *NOXO1* gene promoter upon stimulation of cells with TNF-α (Fig. 2f). These results show that NF-κB is responsible for induction of *NOXO1* expression in TNF-α-stimulated gastric cancer cells.

### Requirement of NOXO1 in TNF-α-induced ROS production

**Fig. 3:**
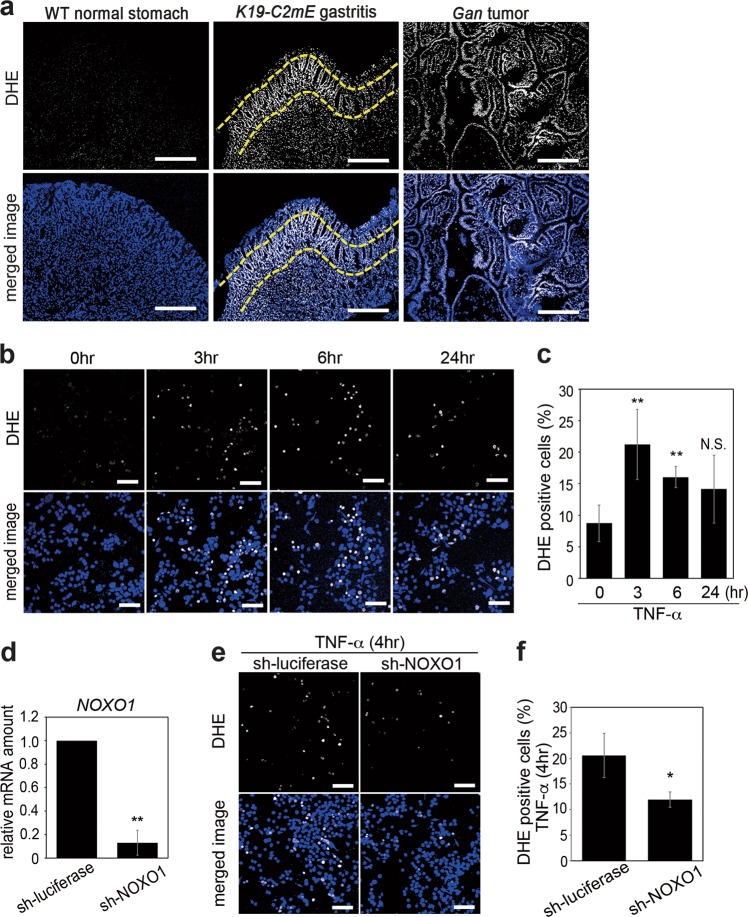
The role of NOXO1 in TNF-α-induced ROS production. **a** Representative photographs of DHE staining (top) and merged images with DAPI staining (bottom) of wild-type mouse stomach (left), *K19-C2mE* mouse gastritis-associated hyperplasia (center), and *Gan* mouse gastric tumors (right). Dashed lines indicate the neck region of *K19-C2mE* hyperplasia. Scale bars, 250 µm. **b** Representative photographs of DHE staining (top) and merged images with DAPI (bottom) of SNU601 cells at the indicated time after TNF-α stimulation. Scale bars, 100 µm. **c** The mean ratio of the SNU601 cells positively stained for DHE at the indicated time after TNF-α stimulation (mean ± s.d.). **p < 0.01; N.S. not significant. **d** The relative mRNA levels of *NOXO1* in the sh-NOXO1 lentivirus-infected cells compared to the control SNU601 cells are shown. **p < 0.01. **e** Representative photographs of DHE staining (top) and merged images with DAPI (bottom) of TNF-α-stimulated sh-NOXO1 lentivirus-infected (right) and TNF-α-stimulated control lentivirus-infected SNU601 cells (left). Scale bars, 100 µm. **f** The mean ratio of the TNF-α-stimulated sh-NOXO1 and control lentivirus-infected cells positively stained for DHE (mean ± s.d.). *p < 0.05

To examine whether ROS production is induced in gastritis and gastric tumors according to the *Noxo1* expression, we performed Dihydroethidium (DHE) staining using frozen sections of stomach tissues of *K19-C2mE* and *Gan* mice that developed gastritis and gastric tumors, respectively. We detected significantly higher ROS activity in the epithelia of both gastritis and gastric tumor tissues, while DHE-positive cells were rarely found in wild-type mouse normal stomach (Fig. 3a). The DHE staining intensity appeared to be stronger in the gland neck-proliferating zone of *K19-C2mE* stomach, which was consistent with the *Noxo1* expression pattern (Fig. 1e). We next examined whether TNF-α signaling increases the ROS level in gastric cancer cells by induction of *NOXO1* expression. Notably, the number of DHE-positive cells was increased significantly after TNF-α stimulation for 3 and 6 h in both SNU601 and SNU719 cells (Fig. 3b, c, Supplementary Fig. 2c). Furthermore, TNF-α-induced increase in the ROS activity was significantly suppressed when the *NOXO1* expression was inhibited by shRNA (Fig. 3d–f). These results indicate that induction of *Noxo1*/*NOXO1* expression is critical for ROS production in the gastritis and gastric tumors.

### Suppression of epithelial proliferation by NOX inhibitor treatment

**Fig. 4:**
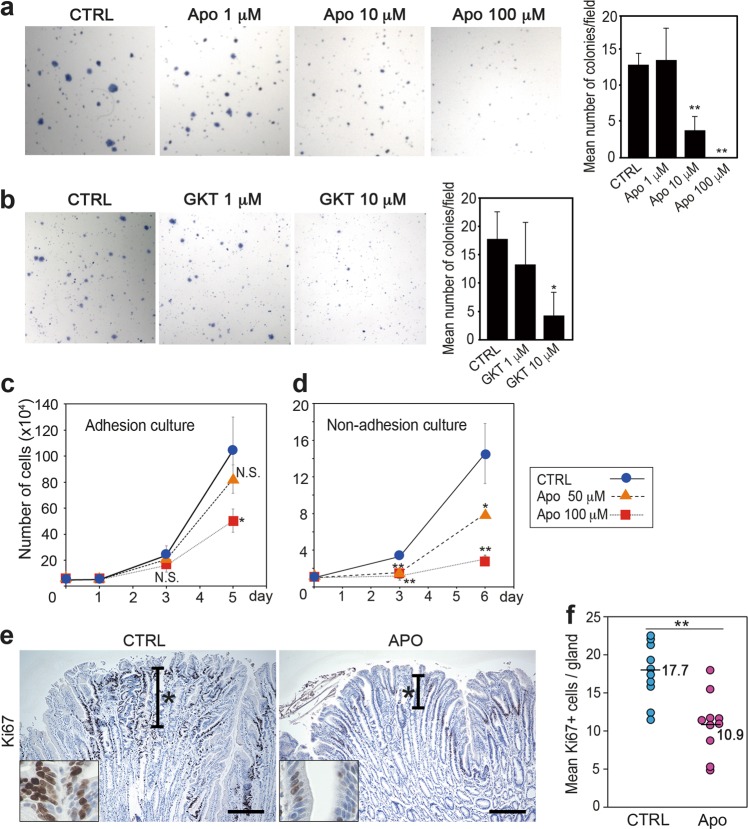
The suppression of cell proliferation by treatment with a NOX inhibitor. **a** Representative photographs of soft agar colonies of MKN45 cells treated with the NOX inhibitor, apocynin (Apo). The mean numbers of soft agar colonies of apocynin-treated (Apo) and control MKN45 cells (CTRL) are shown (mean ± s.d.) in the right. **p < 0.01. **b** Representative photographs of soft agar colonies of MKN45 cells treated with the NOX1/4 inhibitor, GKT136901 (GKT). The mean numbers of soft agar colonies of GKT-treated (GKT) and control MKN45 cells (CTRL) are shown (mean ± s.d.) in the right. *p < 0.05. **c**, **d** Cell proliferation assays of apocynin-treated (Apo) and control MKN45 cells (CTLR) cultured under adhesion (**c**) or non-adhesion conditions (**d**) are shown (mean ± s.d.). *p < 0.05; **p < 0.01; N.S. not significant. **e** Representative photographs of immunohistochemistry for Ki67 in the control *K19-C2mE* (left, CTRL) and apocynin-treated *K19-C2mE* (right, Apo) gastritis-associated hyperplasia. The insets indicate enlarged images. Asterisks indicate proliferating zones. Scale bars, 200 µm. **f** The numbers of Ki67-positive cells per gland in the control (CTRL) and apocynin-treated (Apo) *K19-C2mE* mouse gastric hyperplastic lesions. Bars indicate mean values. **p < 0.01

We next examined whether NOX1 promotes stomach tumorigenesis by inhibitor treatment. We previously showed that suppression of *NOXO1* expression by siRNA resulted in decreased colony formation of gastric cancer cells [13]. Consistently, treatment of MKN45 cells with the NOX inhibitor apocynin or the NOX1/4-specific inhibitor GKT136901 resulted in significant suppression of the soft agar colony formation in a dose-dependent mechanism (Fig. 4a, b). These results suggest that NOX1-dependent ROS signaling has a role in tumorigenicity. Importantly, apocynin treatment significantly suppressed MKN45 cell proliferation (Fig. 4c), and the proliferation rate was more severely suppressed when cells were cultured under non-adhesive conditions (Fig. 4d). Signaling from the extracellular matrix to adhesive cultured cells may contribute to NOX1/ROS-induced cell proliferation, although this point remains to be examined. We next performed a cell cycle analysis using MKN45 cells. The Sub-G1 fraction was significantly increased among apocynin-treated cells, suggesting that NOX inhibition induces apoptosis, which may contribute to the decreased proliferation rate (Supplementary Fig. 3).

Since *Noxo1* is induced and ROS activity is increased in proliferating epithelial cells of non-tumorous gastritis-associated hyperplastic lesions, we treated *K19-C2mE* mice with apocynin to examine the proliferation. Importantly, the mean number of Ki67-positive proliferating cells in hyperplastic mucosa decreased significantly in the apocynin-treated mice, declining to ~60% of that of control mice (Fig. 4e, f). Accordingly, NOX1-induced ROS signaling has a possible role in increased proliferation of epithelial cells of gastritis and gastric cancer.

### SOX2 activation in gastric cancer cells by NOX and ROS pathway

**Fig. 5:**
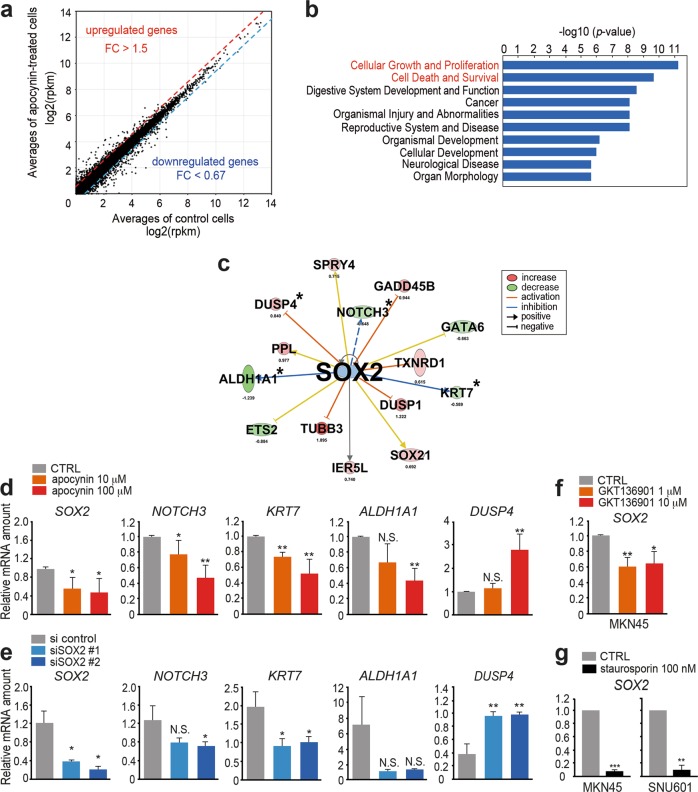
The suppression of the SOX2 pathway by NOX inhibition in vitro. **a** The results of RNAseq are shown as an expression plot of each gene in apocynin-treated and control MKN45 cells. The average log2 (rpkm) values of two trials were plotted. **b** The results of a GO term analysis for “disease and bio function” using the differentially expressed genes in apocynin-treated MKN45 cells. **c** Depicted results of an ingenuity pathway analysis (IPA) for SOX2 and its targets with an increased or decreased expression in apocynin-treated MKN45 cells are shown. **d** The relative mRNA levels of *SOX2* and *SOX2*-target genes (indicated by asterisks in (**c**)) in the apocynin-treated and control (CTRL) MKN45 cells are shown (mean ± s.d.). *p < 0.05; **p < 0.01; N.S. not significant. **e** The relative mRNA levels of *SOX2* and *SOX2*-target genes (indicated by asterisk in (**c**)) in the *SOX2*-targeted siRNA-transfected (siSOX2#1 and #2) and control (si control) MKN45 cells are shown (mean ± s.d.). *p < 0.05; **p < 0.01; N.S. not significant. **f** The relative mRNA levels of *SOX2* in the GKT136901-treated and control (CTRL) MKN45 cells are shown (mean ± s.d.). *p < 0.05; **p < 0.01. **g** The relative mRNA levels of *SOX2* in the staurosporin-treated MKN45 and SNU601 cells and no-drug control (CTRL) are shown (mean ± s.d.). **p < 0.01; ***p < 0.01

To examine the molecular mechanisms underlying NOX1-induced cell proliferation, we performed an RNAseq analysis of apocynin-treated and untreated MKN45 cells (Fig. 5a). We extracted 309 differentially expressed genes that showed increase (>1.5-fold) or decrease (<0.67-fold) in apocynin-treated cells compared with untreated control cells. A GO term analysis using these 309 genes revealed that “cellular growth and proliferation” and “cell death and survival” pathways were significantly affected by apocynin treatment (Fig. 5b), supporting the notion that NOX-dependent ROS signaling accelerates proliferation and survival of gastric epithelial cells.

We next performed an ingenuity pathway analysis (IPA) using the RNAseq results of apocynin-treated MKN45 cells and *K19-C2mE* mouse gastritis tissues (Supplementary Table 2). Notably, among significantly activated pathways in *K19-C2mE* mouse gastritis compared with normal stomach, the SOX2 pathway was downregulated in apocynin-treated MKN45 cells, indicating that SOX2 is a possible target of NOX1/ROS pathway in gastritis tissues (Fig. 5c). We confirmed that the expression level of *SOX2* and SOX2-target genes *NOTCH3*, *KRT7*, and *ALDHA1* was significantly decreased in MKN45 cells by apocynin treatment, while that of *DUSP4*, a negatively regulated SOX2-target gene, was increased (Fig. 5d). The apocynin-induced downregulation of *SOX2* and SOX2-target genes was also observed in both SNU601 and SNU719 cells (Supplementary Fig. 4). Furthermore, similar changes in expression were confirmed when the SOX2 expression was suppressed by the transfection of siRNA for *SOX2* to MKN45 cells (Fig. 5e). We further confirmed that the *SOX2* expression was significantly decreased in MKN45 cells by GKT136901 treatment (Fig. 5f). Moreover, treatment of MKN45 and SNU601 cells with the protein kinase C (PKC) inhibitor staurosporin caused a significant decrease in the *SOX2* expression (Fig. 5g). It has been shown that NOX1 activation is regulated by PKC-induced NOXO1 phosphorylation [15]. Taken together, these results indicate that NOX1/ROS pathway induces increase of SOX2 expression in gastric tumor cells. SOX2 expression labels normal adult gastric stem cells, and SOX2-positive epithelial cells are a potent origin of gastric cancer [23, 24]. Accordingly, NOX1/ROS signaling may promote an increase in SOX2-exressing undifferentiated epithelial cells, contributing to the development of gastritis-associated hyperplasia and gastric tumors.

### SOX2 activation in gastric hyperplasia by the NOX/ROS pathway

**Fig. 6:**
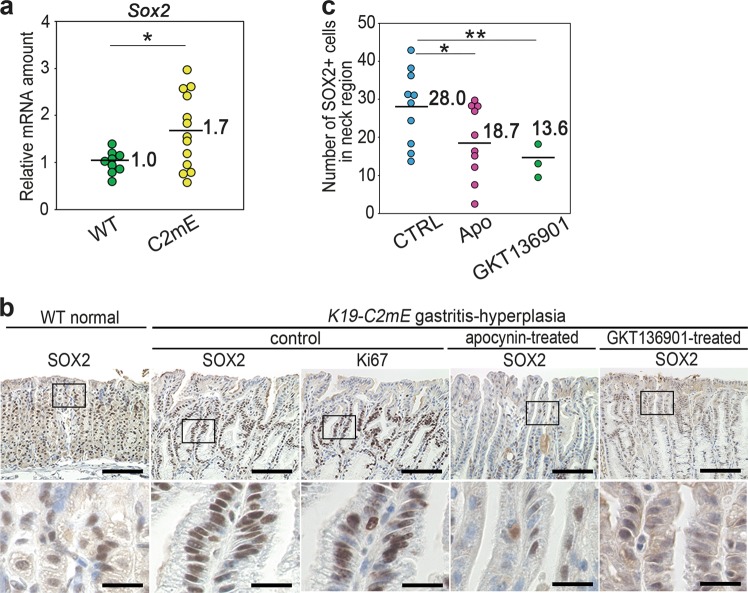
The suppression of the SOX2 expression by NOX inhibition in vivo. **a** The relative mRNA levels of *Sox2* in *K19-C2mE* mouse gastritis-associated hyperplasia (C2mE) and wild-type (WT) mouse stomach to the mean WT value. *p < 0.05. **b** Representative photographs of immunohistochemistry for SOX2 and Ki67 in control, apocynin-treated and GKT136901-treated *K19-C2mE* mouse gastritis-associated hyperplasia and wild-type normal stomach (WT). Low-magnification (top) and enlarged images (bottom) of the boxed area on the top are shown. Scale bars, 100 µm (top) and 20 µm (bottom). **c** The mean numbers of cells positively stained for SOX2 in the gland neck (proliferating zone) in apocynin-treated (Apo), GKT136901-treated, and control (CTRL) *K19-C2mE* mice are plotted with indications of the mean value. *p < 0.05; **p < 0.01

We also detected a significant increase in the *Sox2* mRNA level in *K19-C2mE* gastritis tissues in comparison to the wild-type normal stomach level (Fig. 6a). In the wild-type normal stomach, the SOX2 expression is limited to the small isthmal cells in the gland neck (Fig. 6b). Moreover, the number of SOX2-expressing cells increased and clustered in the gland neck of hyperplasic lesions of *K19-C2mE* mouse gastritis, where Ki67-positive cells were localized (Fig. 6b). In contrast, the number of SOX2-expressing cells was significantly decreased by treatment of mice with apocynin or GKT136901, suggesting that NOX1/ROS signaling has a role in the SOX2-positive cell expansion in inflammation-associated gastric hyperplasia (Fig. 6b, c).

### The role of Noxo1 in gastritis-associated metaplastic hyperplasia

**Fig. 7:**
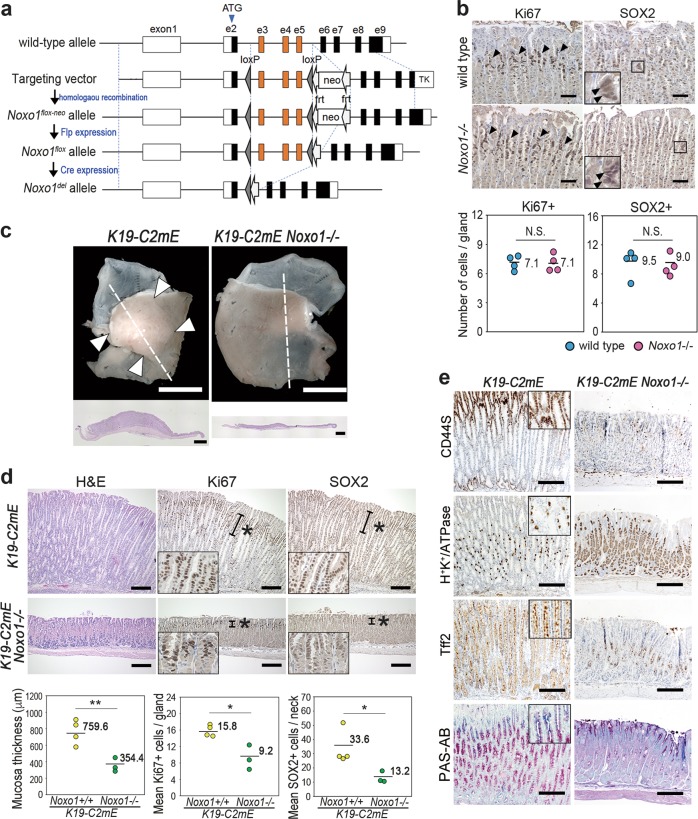
The suppression of gastric metaplastic hyperplasia by *Noxo1* gene disruption. **a** A gene-targeting strategy for the disruption of the *Noxo1* gene by homologous recombination to excise exons 3–5 by Cre-induced recombination. *Noxo1*^*del*^ indicates the targeted allele in mice. **b** Representative immunohistochemistry of Ki67 (left) and SOX2 (right) in the glandular stomach of wild-type mouse (top) and *Noxo1*−/− mouse (bottom). The insets are enlarged images of the boxed areas, and arrowheads indicate SOX2-positive cells. Scale bars, 100 µm. The mean numbers of Ki67-positive cells (left) and SOX2-positive cells (right) per gastric gland of wild-type and *Noxo1*−/− mice plotted with the mean value are shown on the bottom. N.S. not significant. **c** Representative macroscopic photographs of *Noxo1* wild-type and *K19-C2mE Noxo1*−/− mouse stomach at 14 weeks of age (top). Scale bars, 5 mm. Arrowheads indicate gastric hyperplasia in *K19-C2mE* mice. Low-powered histology images of the entire glandular stomach corresponding to the dashed lines are shown (bottom). Scale bars, 1 mm. Note that hyperplastic lesions were not found in the *K19-C2mE Noxo1*−/− glandular stomach. **d** Representative photographs of H&E staining and immunohistochemistry for Ki67 and SOX2 in *K19-C2mE* *Noxo1* wild-type (top) and *K19-C2mE Noxo1*−/− mouse glandular stomach (bottom). Asterisks indicate proliferating zones. Insets indicate enlarged images. Scale bars, 200 µm. The mean mucosal thickness and the number of Ki67-positive cells and SOX2-positive cells were scored and shown with the mean value in the bottom. *p < 0.05; **p<0.01. **e** Representative photographs of immunohistochemistry for *K19-C2mE* (left) and *K19-C2mE Noxo1*−/− mouse glandular stomach (right). Insets indicate enlarged images. Scale bars, 200 µm

To further examine the role of *Noxo1* induction and subsequent NOX1 complex activation in gastric phenotypes, we constructed conventional *Noxo1* gene knockout mice that carried the *Noxo1*^*del*^ allele by homologous recombination (Fig. 7a). Disruption of the *Noxo1* gene did not cause any morphological changes in gastric mucosa, and the Ki67-positive and SOX2-positive cell numbers in *Noxo1*−/− mouse stomach were similar to wild-type mouse level (Fig. 7b), indicating that Noxo1 is unnecessary for survival and proliferation of normal stomach epithelia.

We next crossed *Noxo1*−/− mice with *K19-C2mE* mice, and generated *K19-C2mE Noxo1*−/− double mutant mice. Importantly, the development of hyperplasia in the stomach of *K19-C2mE* mice was significantly suppressed by *Noxo1* gene disruption (Fig. 7c). Histologically, the mean gastric mucosal thickness and the number of proliferating cells were decreased significantly in *K19-C2mE Noxo1*−/− mice in comparison to *Noxo1*+/+ *K19-C2mE* mouse levels (Fig. 7d). It has been shown that NOX1 pathway regulates activation of NF-κB [25, 26]. We therefore examined whether NF-κB is suppressed by *Noxo1* gene disruption in gastric epithelial cells. Notably, TNF-α stimulation caused increased expression of TNF-α in the *K19-C2mE Noxo1*−/− mouse-derived gastric epithelial cells at a level similar to the *Noxo1*+/+ cells, suggesting that TNF-α can activate NF-κB in *Noxo1*-disrupted cells (Supplementary Fig. 5). These results also rule out the possibility that NF-κB inhibition suppressed hyperplasia in *K19-C2mE Noxo1*−/− mice.

Importantly, the SOX2-expressing cells were limited to the isthmus of the *K19-C2mE Noxo1*−/− mouse gastric mucosa, and the mean number of SOX2-expressing cells was significantly lower than in *Noxo1* wild-type mouse stomach (Fig. 7d). In the *K19-C2mE Noxo1*−/− gastric epithelial cells, *Krt7* expression level was significantly decreased, while *Dusp4* level was increased, suggesting that *Noxo1* disruption suppresses the SOX2 pathway in gastric epithelial cells (Supplementary Fig. 6). Although the expression of other SOX2-target genes (*Notch3* and *Aldha1*) did not change by *Noxo1* disruption, this may be caused by the cell context-dependent regulation of SOX2. In addition, the numbers of CD44-positive undifferentiated epithelial cells were decreased, while those of H^+^K^+^/ATPase-expressing differentiated cells were increased in *K19-C2mE Noxo1*−/− mice (Fig. 7e). Mucous cell metaplasia consisting of TFF2-positive epithelial cells was suppressed in *K19-C2mE Noxo1*−/− mouse stomach. Accordingly, Noxo1 induction and NOX1/ROS signaling activation may induce gastric metaplastic hyperplasia in the inflamed mucosa via the expansion of undifferentiated cells.

## Discussion

*H. pylori* infection-associated inflammatory response induces oxidative stress in gastric mucosa, which has an important role in triggering tumorigenesis in the stomach [10, 27]. NOX family members have shown to produce ROS in tumor tissues [16]. We found here that NOX1 complex components are upregulated significantly in the stomach by an inflammation-dependent mechanism. We showed that *NOXO1* expression is directly regulated by transcription factor NF-κB under TNF-α signaling (Supplementary Fig. 7). Since the activation of NOX1 complex is regulated by the protein level and phosphorylation of NOXO1 [15, 28], NF-κB is possibly important for NOX1/ROS pathway activation both in the gastritis and gastric tumors. ROS signaling has been shown to be capable of activating NF-κB [16], suggesting a positive feedback loop of NOX1/ROS signaling and NF-κB in the inflamed gastric mucosa. Notably, NF-κB activation is reportedly important for acquisition of stemness through dedifferentiation of intestinal epithelial cells [29], and the cooperation of the NF-κB and NOX1 signaling facilitate cell proliferation of both intestinal stem cells and cancer cells [20]. These results suggest that the activation of a NOX1/ROS and NF-κB feedback loop is important for tumor development by acquisition and maintenance of stem cell characteristics.

Although TNF-α is a major proinflammatory cytokine that activates NF-κB, innate immune response through Toll-like receptor (TLR) signaling also activates NF-κB pathway through MyD88 activation [30], enabling the regulation of NOX1/ROS signaling. We previously reported that the *Myd88* gene knockout in gastric tumor mouse model significantly suppressed gastritis and gastric tumor development with the suppression of NF-κB activity, indicating that TLR signaling through NF-κB has a role in tumorigenesis [31]. It has been reported that TLR2 signaling through MyD88 is also required in Stat3-driven gastric tumorigenesis [32, 33]. Furthermore, the TLR2/MyD88 pathway is essential for maintaining normal intestinal stem cells and intestinal tumorigenesis [34]. Accordingly, the activation of the NOX1/ROS signaling is also possibly responsible for TLR/MyD88 signaling-induced stemness and tumorigenesis in gastric mucosa. Consistently, *Noxo1* is listed as an upregulated gene in normal mouse gastric stem cells [13, 35], supporting the notion that NOX1/ROS signaling helps maintain gastric stem cells.

While the mechanisms underlying the mediation of cell proliferation and survival by NOX1/ROS signaling remain unclear, NOX-derived ROS signaling activates the autophosphorylation of receptor tyrosine kinase (RTK) by inactivating protein tyrosine phosphatase (PTPs) [16]. The oxidative modification of cysteine residues that are sensitive for redox stimulation in the catalytic domains of PTPs results in the hyperphosphorylation of RTK and activation of downstream oncogenic signaling. In *Gan* mouse gastric tumors, PGE_2_ signaling through EP4 receptor activates signaling through epidermal growth factor receptor (EGFR), which causes promotion of gastric tumorigenesis [36]. Approximately 30–40% of human gastric cancers show amplification of RTK genes, including EGFR [10, 21], and EGFR expression is associated with poor prognosis of patients with gastric cancers [37]. In this study, we showed that disrupting the *Noxo1* gene significantly suppressed COX-2/PGE_2_ pathway-induced gastric hyperplasia. Accordingly, NOX1/ROS signaling may contribute to the activation of EGFR signaling by suppressing PTP functions, which may be a major tumor-promoting mechanism of NOX1/ROS signaling (Supplementary Fig. 7).

We found that SOX2-expressing cells were increased significantly by NOX1/ROS pathway activation in gastritis-associated hyperplastic mucosa. Although SOX2 has paradoxically had a tumor-suppressor role in gastric tumorigenesis, it marks normal gastric stem cells that can be a potent origin of gastric cancer [23, 24]. Accordingly, the positive feedback loop of the NOX1/ROS pathway and NF-κB may contribute to hyperplastic changes of gastric mucosa to form preneoplastic lesions by expanding SOX2-positive undifferentiated epithelial cells (Supplementary Fig. 7). We failed to determine whether SOX2 has a functional role in tumorigenesis; however, our findings indicate that inflammation-associated SOX2 expression can be a useful marker for detecting an early stage of gastric cancer.

In conclusion, we found that NOX1 complex components are upregulated in epithelial cells of both gastritis and gastric tumors, indicating the inflammation-dependent activation of NOX1/ROS signaling in the stomach. Furthermore, NOX1/ROS signaling causes the expansion or increased proliferation of SOX2-expressing stem cells and undifferentiated cells in the stomach, contributing to development of preneoplastic lesion in the stomach. Therefore, targeting NOX1 complex or downstream ROS signaling in addition to *H. pylori* eradication may be a potential strategy for prevention of gastric cancer.

## Materials and methods

### Mouse experiments

*K19-C2mE* and *Gan* mice were previously described [7, 38]. In brief, *K19-C2mE* transgenic mice develop metaplastic hyperplasia that are associated with inflammation by expression of *Ptgs2* and *Ptges*, while *Gan* mice show gastric tumor development by expression of *Ptgs2*, *Ptges* and *Wnt1*. *K19-C2mE* mice and C57BL/6 wild-type mice were treated with apocynin (Sigma) in drinking water (80 µg/ml) for 10 weeks from 5 weeks of age or injected intraperitoneally with GKT136901 (AOBIOUS, Gloucester, MA, USA) at 40 mg/kg/day for 3 weeks (*n* = 10 and 3, respectively). The construction of *Noxo1* knockout mice is described in Supplementary Materials and Methods. Compound mutants *K19-C2mE Noxo1−/−* and control *K19-C2mE Noxo1*+/+ (*n* = 3 for each) were examined at 14 weeks of age. The protocol for all animal experiments were approved by the Committee on Animal Experimentation, Kanazawa University, Japan.

### Real-time reverse transcription-polymerase chain reaction (RT-PCR)

For expression analysis, total RNA was prepared from the stomach of *Gan* (*n* = 9), *K19-C2mE* (*n* = 13), and wild-type mice (*n* = 9) using ISOGEN (Nippon Gene, Tokyo, Japan). For SOX2-target gene analysis using *K19-C2mE Noxo1*−/− and control mice, gastric epithelial cells were separately collected from frozen tissues by laser microdissection (LMD) on LMD7000 (Leica Microsystems, Wetzlar, Germany). For human tissues, gastric tumors and normal stomach (*n* = 24) were collected at Kanazawa University Hospital. The protocol for experiment using clinical samples was approved by the Kanazawa University Medical Ethics Committee. At the tissue collection, written informed consent was obtained. Total RNA was reverse-transcribed, then PCR-amplified by SYBR Premix ExTaqII (Takara, Kusatsu, Japan) using an AriaMX Real-Time PCR system (Agilent Technologies, Santa Clara, CA, USA). The PCR primers were purchased from Takara or designed by Universal Probe Library (Roche Diagnostics, Indianapolis, IN, USA). The designed primer sequences are indicated in the Supplementary Materials and Methods.

### Human gene expression data analyses

The reads per kilobase per million mapped reads (rpkm) of human gastric cancer RNAseq data (level 3) were downloaded from a public database [21]. The expression data of intestinal-type (*n* = 170) as well as diffuse-type gastric cancer (*n* = 65) with matched non-malignant stomach tissues (*n* = 33) in S11.1a Master Patient Table (https://tcga-data.nci.nih.gov/docs/publications/stad_2014/) were analyzed using the R software program (https://www.R-project.org/).

### Histological analysis and immunohistochemistry

Collected stomach tissues were fixed with paraformaldehyde (4%), then embedded in paraffin blocks. Sections at 4 µm thickness were examined after H&E staining. For immunohistochemistry, antibodies for SOX2 (Cell Signaling, Danvers, MA, USA), Ki67 (Dako, Carpinteria, CA, USA), CD44S (Merck, Darmstadt, Germany), H^+^K^+^/ATPase (MBL, Nagoya, Japan) and TFF2 (Novocastra, Newcastle, UK) were used. Vectastain Elite Kit (Vector Laboratories, Burlingame, CA, USA) was used for visualization of immunostaining. For labeling indices of Ki67 and SOX2, the number of cells with positive staining was counted in more than 20 independent glands, and the mean numbers were calculated.

### In situ hybridization

*Noxo1* mRNA was detected via in situ hybridization by an RNAscope 2.5 HD Assay-Brown (Advanced Cell Diagnostics, Hayward, CA, USA). Tissues were fixed with paraformaldehyde (4%), then they were embedded in paraffin blocks. Sections were retrieved in boiling buffer and treated by protease for 30 min, and in situ hybridization was performed according to the manufacturer’s protocol. DapB was used for a negative control.

### Cell culture experiments

The stomach cancer cells SNU601, SNU719 (Korean Cell Line Bank, Seoul, Korea), MKN45 and Kato-III (Riken Bioresource Center, Tsukuba, Japan) were used for the experiments. They were cultured in RPMI1640 with 10% FBS or under serum-free conditions. 293T cells were obtained from ATCC (Manassas, VA, USA), and were cultured in DMEM 10% FBS. For TNF-α stimulation, cells were serum starved overnight and stimulated by TNF-α at 100 ng/ml (Merck, Kenilworth, NJ, USA). The method for organoid cultures is indicated in the Supplementary Materials and Methods.

For the inhibition of *RELA* and *NOXO1* expression, lentivirus-expressing shRNA was infected. The entry vector pENTRH1 was provided by Dr. Ui-Tei (University of Tokyo, Japan). The CS-RfA-CG, pCAG-HIV-gp, and pCMV-VSV-G-RSV-Rev vectors (Riken Bioresource Center) were used for production of lentivirus. Sequences used for shRNA are indicated in the Supplementary Materials and Methods. To suppress the *SOX2* expression, Silencer^TM^ Select siRNA anti SOX2 (Thermo, s13294, and s13295) was used. Silencer^TM^ Select negative control No.1 siRNA was used as control. The protocol for analysis of cell cycle is shown in the Supplementary Materials and Methods.

For inhibitor treatment, cells were treated with apocynin, a NOX inhibitor (Sigma), NOX1/4-specific inhibitor GKT136901 (AOBIOUS), or PKC inhibitor staurosporin (Wako Chemical, Osaka, Japan) at the indicated concentration.

### Luciferase reporter assay

The *NOXO1* promoter region (positions from −2000 to the transcription starting site) was cloned into pGL4 (Addgene). NF-κB binding sites were predicted using the PROMO 3.0 software program (http://alggen.lsi.upc.es/recerca/frame-recerca.html). pRL-TK (Promega) was used for the control. Reporter plasmids were transfected with using Lipofectamine 2000 (Invitrogen, Carlsbad, CA, USA), and transfected cells were serum starved for 12 h prior to the TNF-α treatment (100 ng/ml). The luciferase activity was determined using Dual Luciferase Reporter Assay System (Promega).

### ChIP assay

The cells were crosslinked by treatment with formaldehyde (Wako). ChIP assay was performed using the EZ-ChIP and ChIPAb + NFκB p65 (RelA) Monoclonal Antibody Set (Milipore). Primer sequences for *NOXO1* promoter are shown in the Supplementary Materials and Methods.

### DHE staining

SNU601 were stimulated with TNF-α, and the cells were stained with DHE (Thermo) for 10 min at 37 °C, fixed with 4% PFA, and mounted with VECTASHIELD with DAPI (Vector Laboratories). Fresh tissues were embedded in Tissue-Tek O.C.T. compound (Sakura, Tokyo, Japan), then frozen tissue sections were prepared and stained with DHE for 7 min at 37 °C and mounted. Staining signals were examined using a TCS SP8 confocal microscope (Leica Microsystems). The mean number of cells with DHE-positive staining was determined by counting five independent microscopy fields.

### Immunoblotting analysis

Cells were lysed in TNE buffer with complete mini protease inhibitor (Roche). The 10 µg of protein samples were separated on a SDS-polyacrylamide gel. The primary antibodies for phosphorylated p65 and p65 (Cell Signaling) and GAPDH (Merck) were purchased. Polyclonal anti-NOXO1 antibody was generated (Scrum, Tokyo, Japan). The ECL detection system (GE Healthcare, Buckinghamshire, UK) was used for detection.

### Soft agar colony formation assay

MKN45 cells (1.0 × 10^4^) were seeded in 6-well plates in 0.4% agar, and cultured for 12 days with or without the NOX inhibitor apocynin (Sigma) or the NOX1/4 inhibitor GKT136901 (AOBIOUS). The cells were stained Giemsa Solution (Wako), and the numbers of colonies per well were counted under a dissecting microscope.

### Next-generation RNA sequencing

MKN45 cells were cultured with or without supplement of 100 µM apocynin (Sigma). Total RNA was extracted at day 4 by ISOGEN (Nippon Gene) and RNAeasy mini kit (Qiagen). Sequence library was made using a TruSeq Stranded mRNA Sample Prep Kit (Illumina) and XT-Auto System (Agilent Technologies, Santa Clara, CA, USA) and analyzed by HiSeq 2500 (Illumina). The sequencing results were deposited in the Gene Expression Omnibus under the accession number GSE111520. Expression of human gastritis and intestinal metaplasia was examined using the database GSE60662. The results of RNA sequences results of mouse models were examined using the previously deposited data with the accession number GSE70135 [31].

### Statistical analysis

All data were examined by *t*-test. *p* values <0.05 were considered as statistically significant.

## Supplementary information

Supplementary Materials and Methods

Supplementary Figure 1

Supplementary Figure 2

Supplementary Figure 3

Supplementary Figure 4

Supplementary Figure 5

Supplementary Figure 6

Supplementary Figure 7

Supplementary Table 1

Supplementary Table 2
