# Supplementary material for: NF-κB-induced NOX1 activation promotes gastric tumorigenesis through the expansion of SOX2-positive epithelial cells
Source: Oncogene. 2019 Jan 30;38(22):4250–63. doi: 10.1038/s41388-019-0702-0 (PMC6756228; doi:10.1038/s41388-019-0702-0)
Supplement: Supplementary file 1 — Supplementary Materials and Methods [file 41388_2019_702_MOESM1_ESM.docx]

Supplementary Materials and Methods

Construction of *Noxo1* conditional knockout mice

BAC clones RPCI-23-124B17, RPCI-23-238L20 and RPCI-23-438P15 (Advanced GenoTechs Co, Tsukuba Japan) containing whole *Noxo1* gene of C57BL/6 mice were used as sources of genomic DNA. A 10 kb genomic DNA fragment including entire *Noxo1* exons and upstream sequences was cloned from BAC into PL253 vector. A PGK-Neo cassette was sandwiched between two FRT sequences and inserted into the intron 5 of *Noxo1* gene. Two loxP sites were inserted before the exon3 and after the exon 5. Thus, exons 3, 4 and 5 of *Noxo1* are deleted when Cre recombinase is expressed. The vector was electroporated to TT2 ES cells, and homologous recombinant clone was selected by G418 screening. Construction of the targeted allele was confirmed by genomic PCR and genomic Southern blotting. Germline transmitted *Noxo1^flox now^* heterozygous mice were crossed with CAG-FLPe transgenic mice (RBRC01834, RIKEN BRC, Japan) to obtain *Noxo1^flox^* mice and subsequently crossed with CAG-Cre transgenic mice (Sakai K, Miyazaki J. *Biochem Biophys Res Commun* 1997; 237: 318-324) to generate conventional *Noxo1* knockout, *Noxo1^del^*, mice. See also Fig. 7a for targeting strategy.

Organoid culture experiments

The primary organoid cultures of *K19-C2mE Noxo1*-/- and *K19-C2mE Noxo1*+/+ mouse gastric epithelial cells were prepared according to the protocol as described (Leushacke, et al. *Nat Cell Biol* 2017; 19: 774-786.). Isolated gastric glands were seeded in Matrigel and cultured in medium supplemented with EGF, Gastrin, FGF10, Noggin, Wnt3 and R-spondin. Rock inhibitor (Y27632, Wako Chemical, Osaka, Japan) and GSK3 inhibitor (CHIR-00921, Tocris Bioscience, Bristol, UK) were added to the medium.

Primer sequences for RT-PCR (designed primers only)

|  | Gene name | Forward primer | Reverse priver |
| --- | --- | --- | --- |
| Human | *NOXO1* | AGATCAAGAGGCTCCAAACG | GGAAGGTCTCCTTGAGGGTCT |
|  | *GAPDH* | GCACCGTCAAGGCTGAGAAC | TGGTGAAGACGCCAGTGGA |
|  | *NOTCH3* | TGCAGCGTGACCGAGATA | CACCCATTATAAATAAAGGAAGACTGA |
|  | *KRT7* | CAGGCTGAGATCGACAACATC | CTTGGCACGAGCATCCTT |
|  | *DUSP1* | CGAGAGGGCTGGTCCTTAT | AAGTCATCACCATAACTGCTTAGAAA |
|  | *DUSP4* | GGATGTCAAGGCGCTGTT | GGCTGTGGTTTCCACAAGA |
|  | *ALDH1A1* | TTTGGTGGATTCAAGATGTCTG | CACTGTGACTGTTTTGACCTCTG |
|  | *SOX2* | TTGCTGCCTCTTTAAGACTAGGA | TAAGCCTGGGGCTCAAACT |
| Mouse | *Noxo1* | CGGCTTCTTTGTACCCAAAC | GGTGTAGGCAGGATCACCAG |
|  | *Notch3* | GACCGTGTGGCCTCTTTC | ATGACACAAGAGGCCTGTCTTC |
|  | *Krt7* | GGCAGCAGCTCGAGACAC | GTCGGTTGATCTCCTCTTCATAC |

Primer sequences for ChIP assay

*NOXO1* promoter Fw: GCAAGAAAGCTGCAAAGGAC

*NOXO1* promoter Rev: GAAAACCCCCTGGGATAGAA

shRNA sequence for lentivirus construction

The sequences for shRNA were designed by siDirect ver 2.0 (http://sidirect2.rnai.jp/)

| Gene | shRNA sequence |
| --- | --- |
| luciferase | 5’-GATTTCGAGTCGTCTTAATGT-3’ |
| *RELA* #1 | 5’-GATGAGATCTTCCTACTGTGT-3’ |
| *RELA* #2 | 5’-GGATTGAGGAGAAACGTAAAA-3’ |
| *RELA* #3 | 5’-GACATTGAGGTGTATTTCACG-3’ |
| *NOXO1* | 5’-GAATTCAGGCAGCTCAAGACC-3’ |

Cell cycle analysis

MKN45 cells were plated in 6 well plates and cultured in the presence or absence of 100 µM apocynin (Sigma). After 3 days, apocynin was additionally added, and cells were trypsinized and fixed using cold ethanol at day 4. Cells were washed and treated with RNase A (0.25 mg/ml) at 37°C for 30min. DNA was stained with propidium iodide (PI) (Sigma) and analyzed by MACSQuant analyzer (Milteny BIotec). At least 5.0X10^3^ cells were analyzed.
