# Supplementary figures and images for: NF-κB-induced NOX1 activation promotes gastric tumorigenesis through the expansion of SOX2-positive epithelial cells
Source: Oncogene. 2019 Jan 30;38(22):4250–63. doi: 10.1038/s41388-019-0702-0 (PMC6756228; doi:10.1038/s41388-019-0702-0)

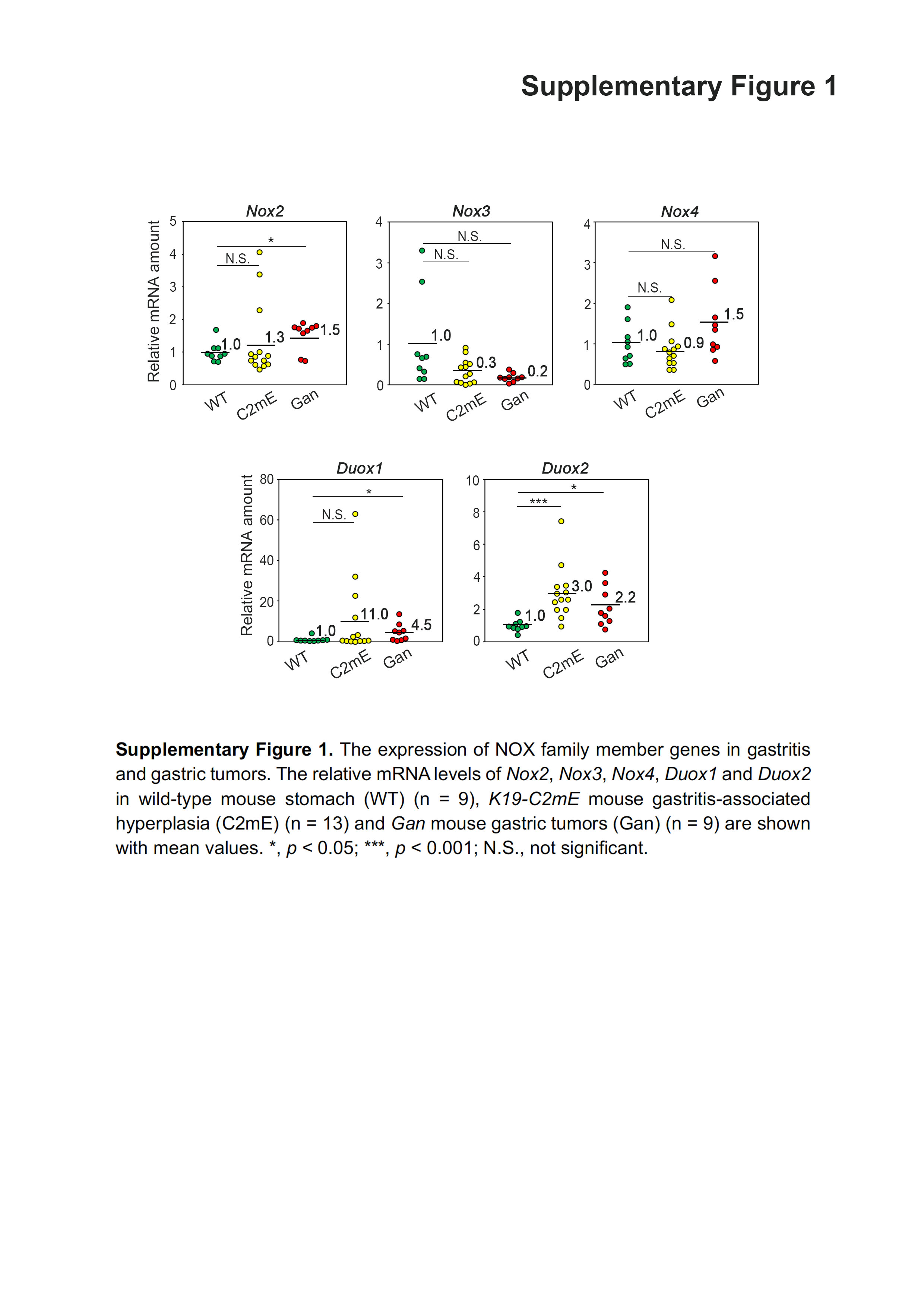

Supplement: Supplementary file 2 — Supplementary Figure 1 [file 41388_2019_702_MOESM2_ESM.jpg]

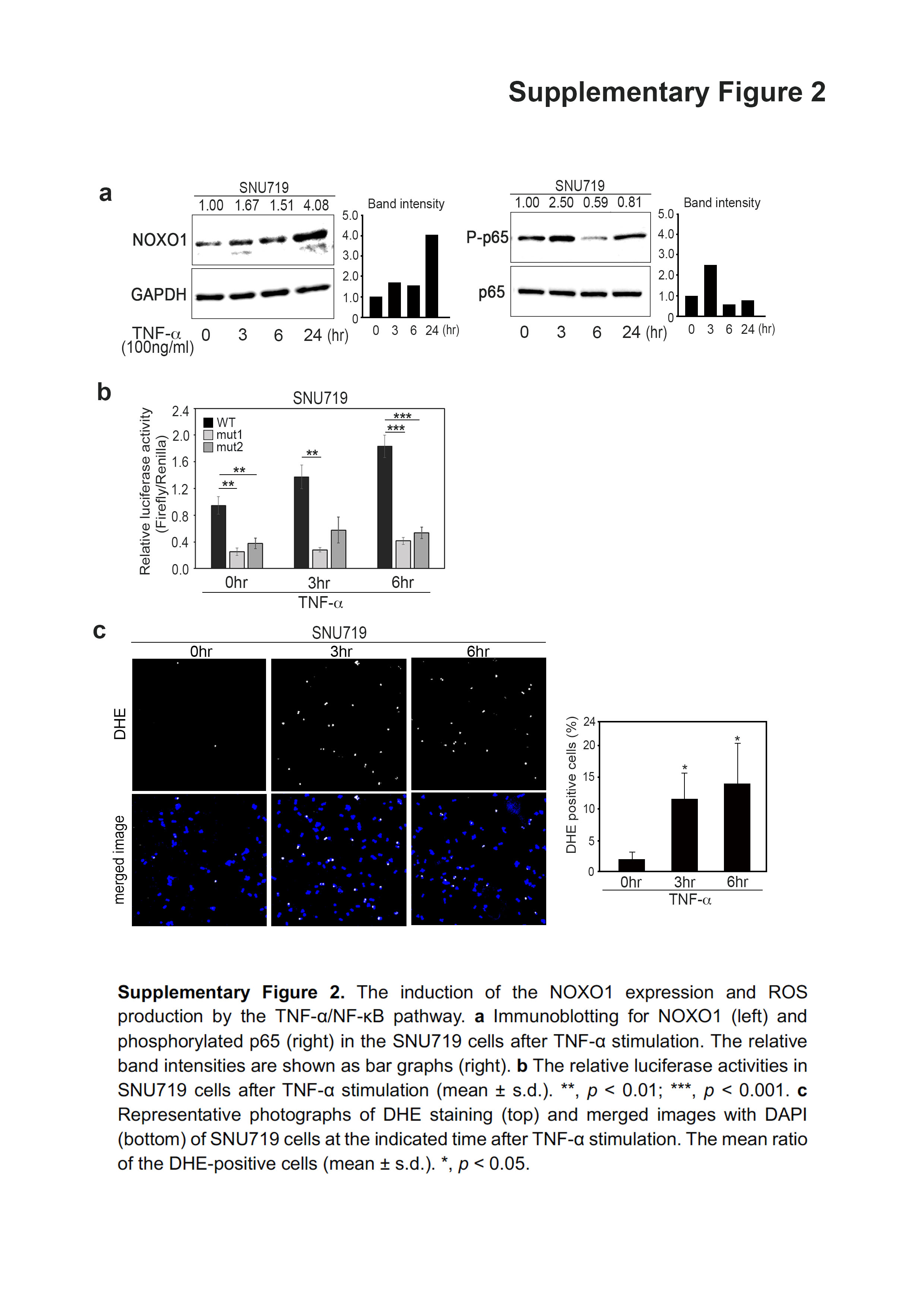

Supplement: Supplementary file 3 — Supplementary Figure 2 [file 41388_2019_702_MOESM3_ESM.jpg]

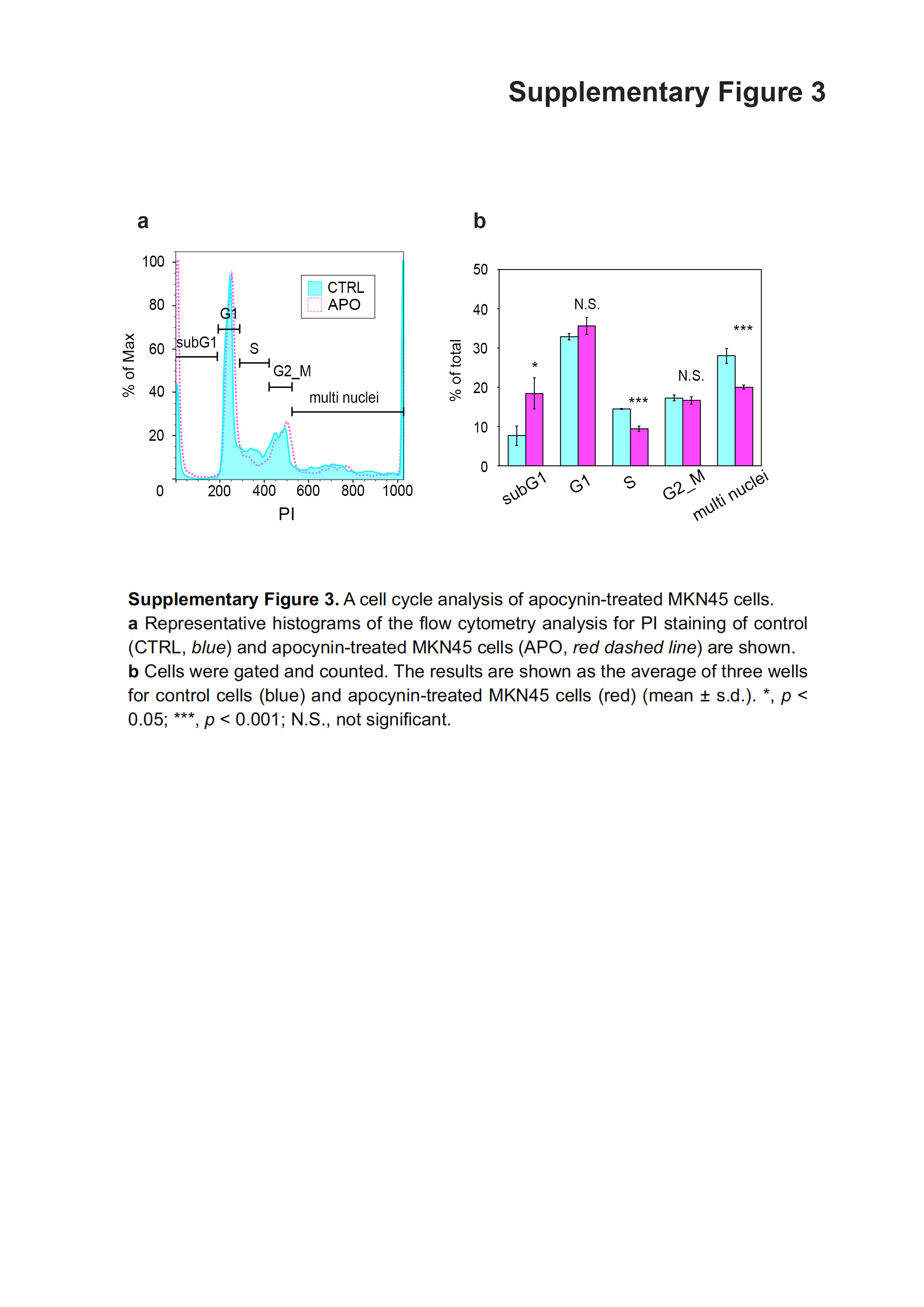

Supplement: Supplementary file 4 — Supplementary Figure 3 [file 41388_2019_702_MOESM4_ESM.jpg]

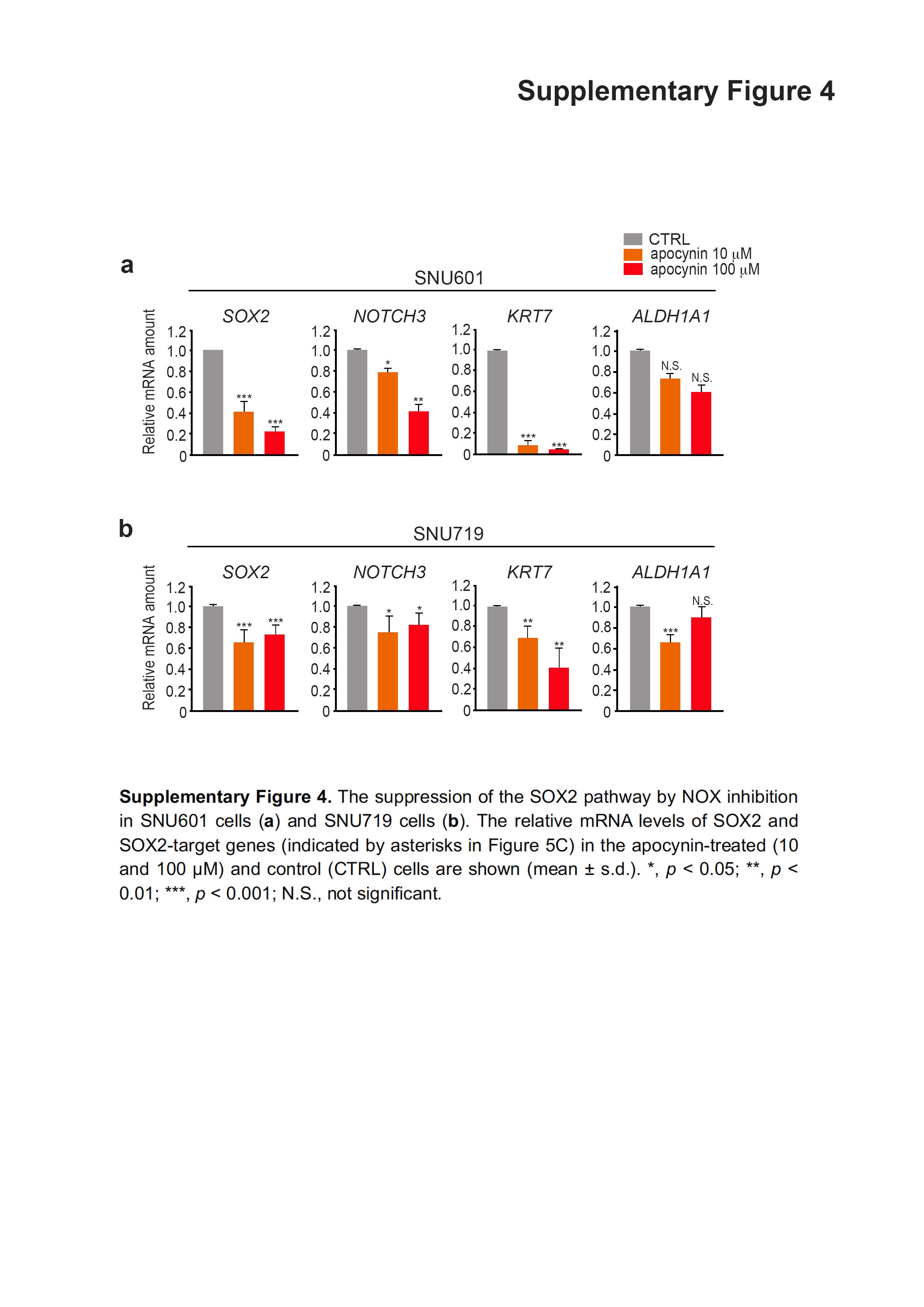

Supplement: Supplementary file 5 — Supplementary Figure 4 [file 41388_2019_702_MOESM5_ESM.jpg]

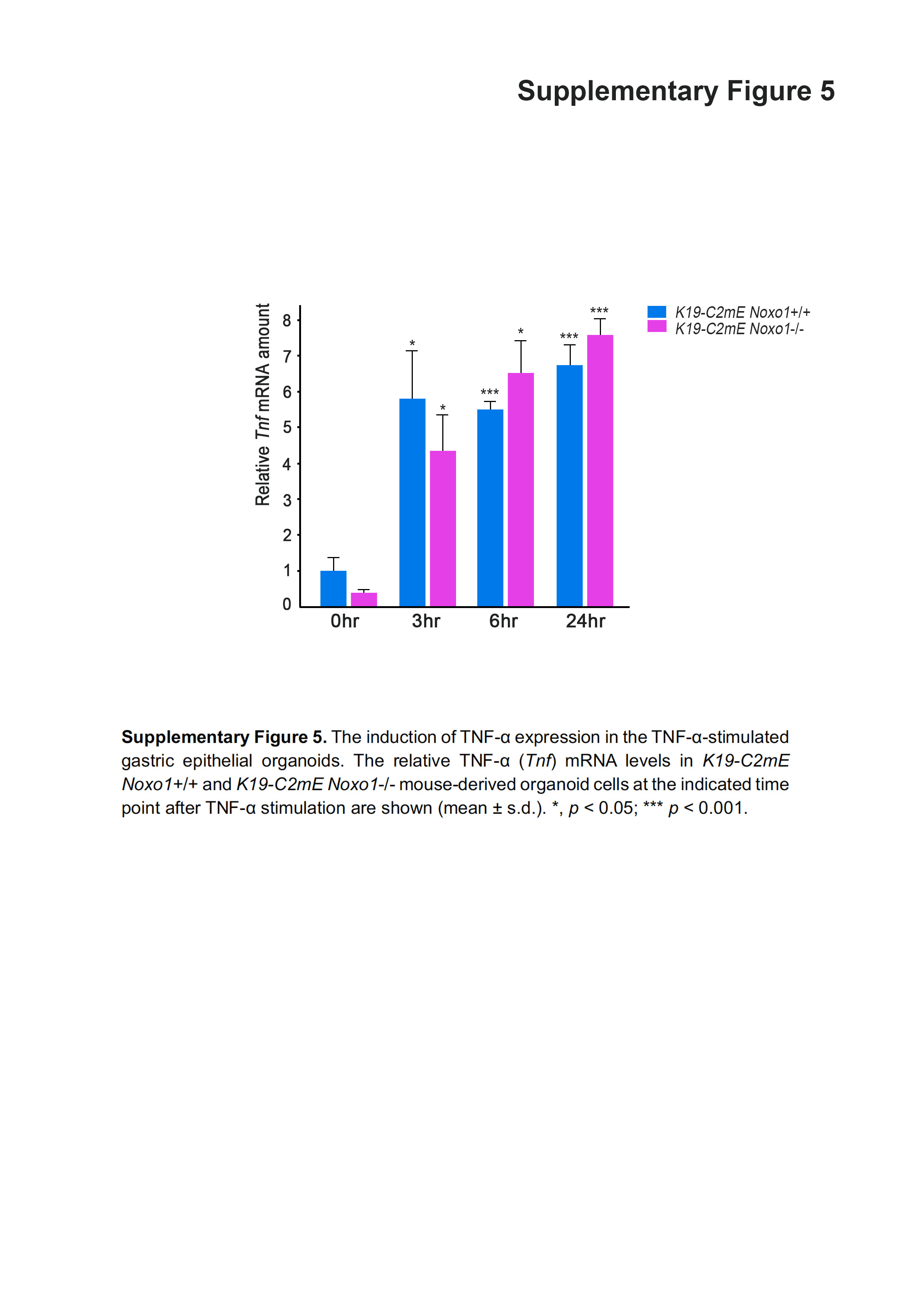

Supplement: Supplementary file 6 — Supplementary Figure 5 [file 41388_2019_702_MOESM6_ESM.jpg]

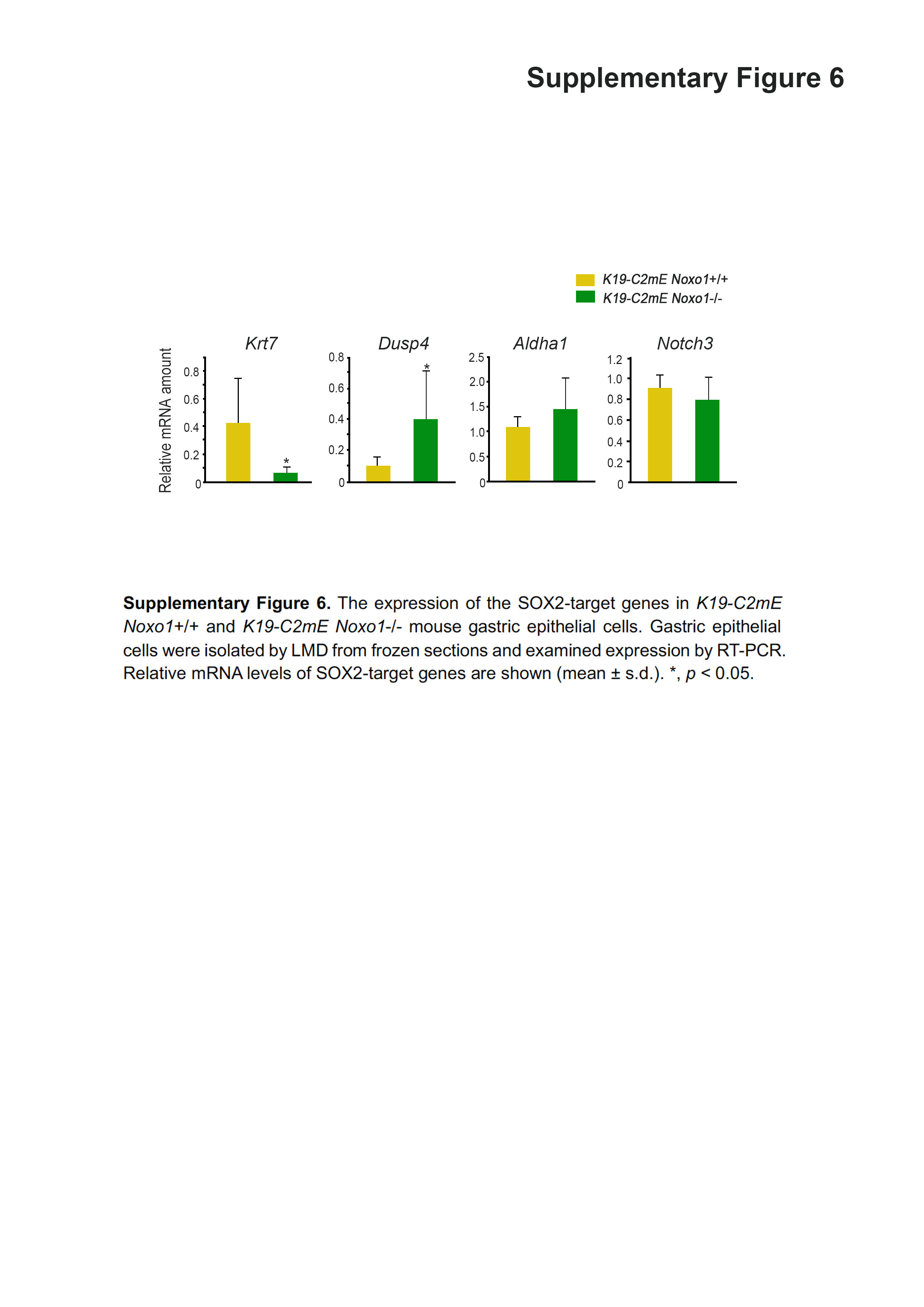

Supplement: Supplementary file 7 — Supplementary Figure 6 [file 41388_2019_702_MOESM7_ESM.jpg]

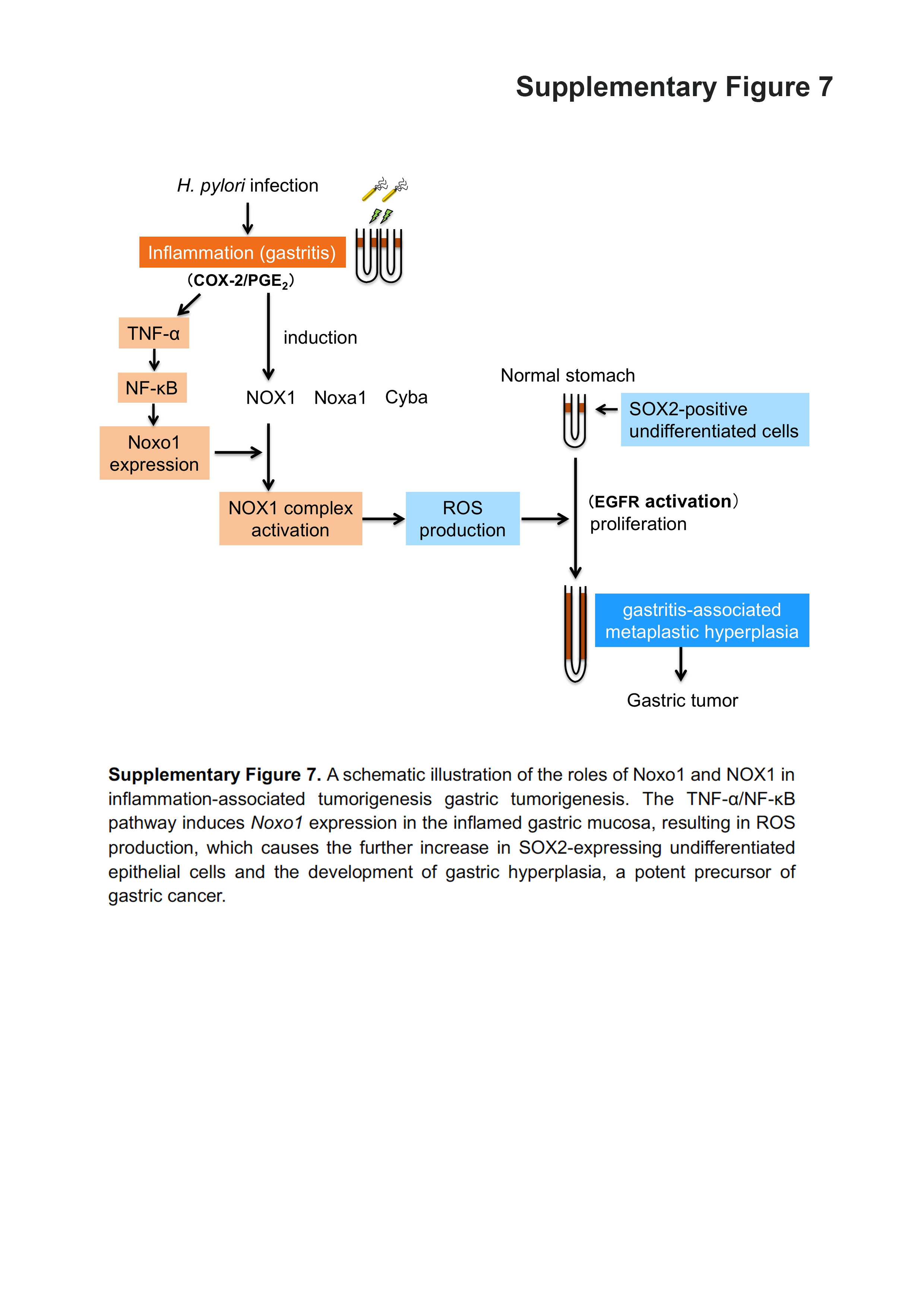

Supplement: Supplementary file 8 — Supplementary Figure 7 [file 41388_2019_702_MOESM8_ESM.jpg]
