# Supplementary material for: NF-κB-induced NOX1 activation promotes gastric tumorigenesis through the expansion of SOX2-positive epithelial cells
Source: Oncogene. 2019 Jan 30;38(22):4250–63. doi: 10.1038/s41388-019-0702-0 (PMC6756228; doi:10.1038/s41388-019-0702-0)
Supplement: Supplementary file 9 — Supplementary Table 1 [file 41388_2019_702_MOESM9_ESM.pdf]

Supplementary Table 1. Activation of NF-κB pathway in gastric lesions.

	human stomach cancer (TCGA)					
	intestinal			diffusive		
Upstream Regulator	Predicted Activation State	Activation z-score	p-value of overlap	Predicted Activation State	Activation z-score	p-value of overlap
NFkB (complex)	Activated[a]	4.841	3.02E-11	Activated	5.089	1.38E-13

	human gastritis (GSE60662)								
	intestinal metaplasia			mild gastritis			severe gastritis		
Upstream Regulator	Predicted Activation State	Activation z-score	p-value of overlap	Predicted Activation State	Activation z-score	p-value of overlap	Predicted Activation State	Activation z-score	p-value of overlap
NFkB (complex)	Activated	2.446	1.11E-04	Activated	5.518	9.32E-19	Activated	5.889	2.42E-13

[a], z-score > 2.0 is considered as significantly activated.
