# Supplementary material for: NF-κB-induced NOX1 activation promotes gastric tumorigenesis through the expansion of SOX2-positive epithelial cells
Source: Oncogene. 2019 Jan 30;38(22):4250–63. doi: 10.1038/s41388-019-0702-0 (PMC6756228; doi:10.1038/s41388-019-0702-0)
Supplement: Supplementary file 10 — Supplementary Table 2 [file 41388_2019_702_MOESM10_ESM.pdf]

Supplementary Table 2. The results of upstream regulator analysis by IPA.

Upstream regulator	K19-C2mE/wild-type[a]		Apo-treated/control[b]	
	z-score	*p*-value	z-score	*p*-value
MYC	4.89	1.53E-19	0.941	3.78E-06
PDGF BB	4.485	1.36E-09	2.658	5.01E-10
TNF	4.462	1.41E-39	0.628	3.17E-05
SOX2	3.615	2.07E-08	-0.505	2.20E-05
PRKCD	1.786	1.45E-02	0.05	4.15E-05
TREM1	1.688	1.24E-10	2.714	7.70E-05
CCND1	1.463	1.26E-02	1.982	2.43E-05
TP63	1.313	4.99E-22	0.714	4.49E-10
CREBBP	1.309	7.17E-09	N.D.	2.45E-05
MGEA5	1.192	3.25E-16	-0.775	9.23E-05
TGFB1	1.176	7.28E-28	2.294	3.78E-05

[a], Upregulated pathways in K19-C2mE mouse gastritis tissues compared with wild-type normal stomach are listed (z-score > 1).

[b], These listed pathways were further examined using differentially expressed genes in apocynin-treated MKN45 cells compared with untreated control cells.
